# Metformin ameliorates maternal high-fat diet-induced maternal dysbiosis and fetal liver apoptosis

**DOI:** 10.1186/s12944-021-01521-w

**Published:** 2021-09-08

**Authors:** Szu-Wei Huang, Yu-Che Ou, Kuo-Shu Tang, Hong-Ren Yu, Li-Tung Huang, You-Lin Tain, I-Chun Lin, Jiunn-Ming Sheen, Chih-Yao Hou, Ching-Chou Tsai, Mao-Meng Tiao

**Affiliations:** 1 Department of Obstetrics and Gynecology, Kaohsiung Chang Gung Memorial Hospital, Chang Gung University College of Medicine, Kaohsiung, Taiwan; 2 Department of Obstetrics and Gynecology, Chiayi Chang Gung Memorial Hospital, Chiayi, Taiwan; 3 Department of Pediatrics, Kaohsiung Chang Gung Memorial Hospital, Chang Gung University College of Medicine, Kaohsiung, Taiwan; 4 Department of Pediatrics, Chiayi Chang Gung Memorial Hospital, Chiayi, Taiwan; 5 Department of Seafood Science, National Kaohsiung University of Science and Technology, Kaohsiung, Taiwan; 6 Graduate Institute of Clinical Medicine, Kaohsiung Medical University, Kaohsiung, Taiwan

**Keywords:** Fatty liver, High-fat diet, Pregnancy, Metformin, Microbiota, Apoptosis

## Abstract

**Background:**

The deleterious effect of maternal high-fat diet (HFD) on the fetal rat liver may cause later development of non-alcoholic fatty liver disease (NAFLD). The aim of this study was to evaluate the effect of maternal HFD-induced maternal hepatic steatosis and dysbiosis on the fetal liver and intestines, and the effect of prenatal metformin in a rat model.

**Methods:**

Sprague–Dawley rats were assigned to three groups (*N* = 6 in each group). Before mating, the rats were randomly assigned to HFD or normal-chow diet (NCD) group for 7 weeks. After mating, the HFD group rats were continued with high-fat diet during pregnancy and some of the HFD group rats were co-treated with metformin (HFMf) via drinking water during pregnancy. All maternal rats and their fetuses were sacrificed on gestational day 21. The liver and intestinal tissues of both maternal and fetal rats were analyzed. In addition, microbial deoxyribonucleic acid extracted from the maternal fecal samples was analyzed.

**Results:**

HFD resulted in maternal weight gain during pregnancy, intrahepatic lipid accumulation, and change in the serum short-chain fatty acid profile, intestinal tight junctions, and dysbiosis in maternal rats. The effect of HFD on maternal rats was alleviated by prenatal metformin, which also ameliorated inflammation and apoptosis in the fetal liver and intestines.

**Conclusions:**

This study demonstrated the beneficial effects of prenatal metformin on maternal liver steatosis, focusing on the gut-liver axis. In addition, the present study indicates that prenatal metformin could ameliorate maternal HFD-induced inflammation and apoptosis in the fetal liver and intestines. This beneficial effect of in-utero exposure of metformin on fetal liver and intestines has not been reported. This study supports the use of prenatal metformin for pregnant obese women.

## Background

The prevalence of maternal obesity is increasing globally. In the USA, the body mass index of approximately 30 % of pregnant women is over 30 kg/m^2^, and it is estimated that more than 21 % of women will be obese globally by 2025 [1]. Maternal obesity will increase the incidence of gestational diabetes and preeclampsia and the risk of neonatal complications such as large-for-gestational-age and admission to the neonatal intensive care unit [1]. Furthermore, several studies have shown that the transfer of excessive fuel to the fetus during gestation increases inflammation and oxidative stress in fetal tissues, leading to morbidities such as obesity, metabolic syndrome, and non-alcoholic fatty liver disease (NAFLD) in the offspring [2, 3].

Maternal obesity has an adverse effect on both, short-term maternal-fetal obstetric outcomes and long-term events such as development of offspring. Therefore, managing maternal obesity is an important issue. The current standard care of non-diabetic maternal obesity is weight loss via surgical or non-surgical methods. Prenatal metformin has been used in pregnant women with type 2 diabetes or gestational diabetes for a long time to improve maternal-fetal obstetric outcomes, and the incidence of fetal anomalies does not increase under in-utero exposure of metformin. However, the American Congress of Obstetricians and Gynecologists does not recommend the use of metformin for non-diabetic obese pregnant women because of limited evidence of benefits [4, 5]. A randomized-controlled trial and a meta-analysis suggested that the administration of prenatal metformin to obese pregnant women without diabetes mellitus reduces the risk of preeclampsia, maternal weight gain during pregnancy, and the incidence of admission to the neonatal intensive care unit [6, 7]. Contrarily, the GRoW trial reported no significant improvement in maternal-fetal outcomes [8].

The aforementioned trials in human were limited to short-term obstetric outcomes and did not evaluate a wide range of metabolic outcomes in offspring, and therefore, the pros and cons of prenatal metformin in maternal obesity could not be assessed comprehensively. Thence, several animal studies have used a model of maternal diet-induced obesity to investigate the effects of prenatal metformin on the fetus or offspring. Desai et al. reported that prenatal metformin administration to high-fat/high-sugar diet-fed Wistar rats decreases inflammatory cytokine levels in fetal plasma and placenta cell lines [9]. Another study using a mouse model revealed that the administration of metformin to maternal mice fed a high-fat diet (HFD) prevents HFD-induced glucose intolerance in the offspring. The study further analyzed offspring gene expression from the liver and subcutaneous adipose tissue and found that intrauterine metformin exposure changes the expression of metabolic genes [10]. Administration of metformin to maternal rats fed high-calorie or high-fructose diets alleviates some diet-induced inflammation and fatty acid changes in fetal hepatic tissues and prevents diet-induced hypertension in the offspring [11, 12].

However, no study has evaluated the influence of prenatal metformin on maternal and fetal metabolism focusing on the gut-liver axis. NAFLD is a chronic liver disease marked by lipid accumulation in the liver; this is strongly associated with obesity and the metabolic syndrome. Recent research suggests that the imbalance of intestinal microbiota, known as dysbiosis, is an important mechanism involved in the pathogenesis of NAFLD [13, 14]. Therefore, in this study, a rat model of HFD-induced NAFLD was established as previously reported [15]. Besides, metformin was administrated to pregnant rats fed HFD. Thereafter, the morphology, apoptosis, and inflammatory state of the maternal and fetal liver and intestines were investigated. Microbial deoxyribonucleic acid (DNA) extracted from the maternal fecal samples was analyzed. We aimed to elucidate the effect of prenatal metformin on maternal hepatic steatosis, intestinal microbiota, and fetal liver and intestinal changes in this HFD-induced NAFLD rat model.

## Methods

### Animals

Animal experiments were conducted in the Animal Experimental Center of Kaohsiung Chang Gung Memorial Hospital. This study was approved by the Institutional Animal Care and Use Committee of the Kaohsiung Chang Gung Memorial Hospital (Approval No. 2,019,052,802; Valid period: 20,191,001 ~ 20,210,930). Sprague-Dawley (SD) rats were housed in the animal facility at the Chang Gung Memorial Hospital, Kaohsiung, Taiwan under a 12-h:12-h light/dark cycle, with lights on at 7:00 am. The pregnant rats were examined for litters at 10:00 am daily.

HFD was purchased from the Research Diets, Inc., New Brunswick, NJ, USA; it consists of 5.56 kcal/g dry weight, 23 g/100 g protein, 35.5 g/100 g carbohydrate, and 35.8 g/100 g saturated fat, mostly in the form of lard (58 kcal% fat D12331, Research Diets). In contrast, the normal-chow diet (NCD) consisted of 3.85 kcal/g dry weight, 19.2 g/100 g protein, 67.3 g/100 g carbohydrate, and 4.3 g/100 g saturated fat. Female rats were arranged to copulate with male rats for 24 h. After copulation, the female rats were housed individually in a cage.

SD rats were randomly allocated into three groups (*N* = 6 for each group):

I. NCD group: 7-week-old female SD rats were fed the normal-chow diet 7 weeks before mating and during gestation. The pregnant rats and their fetuses were sacrificed on gestational day 21 (GD21).
II. HFD group: 7-week-old female SD rats were fed the HFD 7 weeks before mating and during gestation. The pregnant rats and their fetuses sacrificed on GD21.
III. HFMf group: 7-week-old female SD rats were fed the HFD 7 weeks before mating and during gestation. The pregnant rats were administrated metformin (500 mg/kg/day) from gestational day 0. The dose of metformin used here was determined by referring to a previous study [16]. Metformin was dissolved in drinking water (0.55–0.88 g/100 mL) instead of administering via gavage to avoid stress induced by oral gavage in pregnant rats. The dose of metformin was estimated twice weekly during the experimental period based on the body weight of rats and the amount of daily water intake. There were no gastrointestinal adverse effects including diarrhea, vomiting, and reduced appetite during metformin administration. The pregnant rats and their fetuses were sacrificed on GD21.

### Measurement of plasma biochemistry parameters

Blood samples were collected by cardiocentesis [17]. Total cholesterol level and aspartate transaminase (AST) and alanine aminotransferase (ALT) activities were measured using a standard autoanalyzer (Hitachi model 7450; Hitachi, Tokyo, Japan). Blood glucose level in maternal rats was measured using a glucose meter. The blood samples were collected from the tail one day before sacrifice.

### Tissue preparation

Rats were anesthetized with 25 mg/kg Zoletil and 23 mg/kg xylazine on GD21. They were then continuously perfused with normal saline using a peristaltic pump. The liver and small intestine were immediately removed and placed on an ice plate. The lumen of the small intestine was cleaned using ice-cold phosphate buffered saline solution (PBS, pH 7.4). The ileum was embedded in Swiss rolls in paraffin blocks, and then subjected to hematoxylin and eosin (H&E) staining for evaluation of the length of villi and the depth of crypts. The liver tissue was cut into pieces and embedded in paraffin blocks. The rest of the liver and small intestine were dissected and stored at -80 °C. The fetal liver and intestine were also collected for future analyses.

### Western blotting

The liver and small intestine samples were homogenized in a lysis buffer (iNtRON, 17,081; Biotechnology, Seongnam, Korea) and centrifuged at 14,000 × *g* for 5 min at 4℃. The total protein concentration was determined using Bio-Rad Protein Assay Dye Reagent Concentrate (BioRad Laboratories, Inc., Hercules, CA, USA). Protein (65 µg) from the supernatant of each sample was separated by 6–15 % sodium dodecyl sulfate polyacrylamide gel electrophoresis depending on target protein size, and then transferred onto a polyvinylidene difluoride membrane. The membrane was blocked with TBST buffer containing 10 % non-fat milk for 1 h at room temperature. Immunoblotting was performed by incubating the membranes at 4 °C overnight, with specific primary antibodies including tumor necrosis factor-α (TNF-α) antibody (#3707; Cell Signaling, Denver, MA, USA), cleaved-caspase3, phospho-AKT, AKT, Bax antibody (#9661, #9475, #9271, #9272, #2772, #4870; Cell Signaling), Claudin-1 antibody (sc-137,254, sc-166,338; Santa Cruz Biotechnology, Dallas, Texas, USA), Zonula occludens-1 (ZO-1) antibody (67-7300; Thermo Fisher Scientific Inc., Waltham, MA, USA), and Claudin-3 antibody (ab22604, ab15102; Abcam, Cambridge, MA, USA). The membrane was then incubated with secondary horseradish peroxidase-conjugated anti-rabbit antibody (1:5000; Jackson ImmunoResearch, West Grove, PA USA) or anti-mouse antibody (1:10,000; Jackson ImmunoResearch) for 1 h at room temperature. The blots were visualized using an ECL kit (NEL 105001EA; Perlcin Elmer In., Boston, MA, USA).

### Hematoxylin and eosin staining

The tissues of liver and small intestine were fixed in 4 % paraformaldehyde at 4 °C, overnight. The fixed tissues were dehydrated in a gradient series of ethanol —70–100 %, hyalinized in xylene, and embedded in paraffin wax at 55 °C. The formalin-fixed, paraffin-embedded (FFPE) blocks were cut into 4-µm thick sections and were then stained using an H&E Staining Kit (ScyTek Laboratories, West Logan, USA). A Leica DMI 3000 microscope equipped with a digital camera was used for histopathology analysis. The images were quantified for lipid accumulation in the liver using ImageJ (Fiji version 1.8.0) [18].

### Immunohistochemistry: IL-6 staining for inflammation study

The 4-µm sections of FFPE blocks were mounted on silanized slides, deparaffinized in xylene, and rehydrated through serial baths of alcohol to water. The hydrated sections were treated in 3 % hydrogen peroxide for 10 min to eliminate endogenous peroxidase activity and were washed in PBS. Anti-IL-6 antibody (ab6672; Abcam) was diluted to 1:200 in DaVinci Green (Biocare PD900, CA) and the assay was performed using the Ultravision Quanto Detection system HRP DAB kit (TL-060-QHD; Thermo Scientific Inc.). A Leica DMI 3000 microscope equipped with a digital camera was used for observation, and images were quantified using ImageJ (Fiji version 1.8.0) [18].

### Terminal deoxynucleotidyl transferase-mediated deoxyuridine triphosphate biotin nick-end labeling (TUNEL) apoptosis study

The tissues were fixed by incubating in 4 % paraformaldehyde in PBS overnight at 4 °C. The fixed tissues were then paraffin embedded, cut into 4-µm sections, and mounted on slides. An apoptosis detection kit (Roche, 11,684,817,910, Mannheim, Germany) was used following the manufacturer’s instructions. The tissue sections were stained with 3,3′-diaminobenzidine tetrahydrochloride and counterstained with Gill’s hematoxylin. The TUNEL-positive cells were counted in randomly selected high-power fields (200× and 400×) of each section under a light microscope and the proportion of TUNEL-positive cells was calculated. Ten random fields of individual rats were used to analyze the rate of apoptotic cells in the liver and each villus of the ileum.

### Intestinal microbiota analysis

Fecal bacterial DNA was extracted from the cecum of each rat using the QIAamp Fast DNA Stool Mini Kit (QIAamp, Germany). The microbial 16SV3-V4 region was amplified with indexes and adaptor-linked primers. Polymerase chain reaction was conducted using the KAPA HiFi Hotstart PCR kit high-fidelity enzyme in triplicates. Amplicon libraries were quantified using Qubit 2.0 Fluorometer (Thermo Fisher Scientific) and then sequenced on the Illumina HiSeq platform (Illumina, San Diego, US) for paired-end sequencing 250 bp. After removing the singletons and eliminating the chimeras, tags were clustered into operational taxonomic units (OTUs) using USEARCH (v7.0.1090) at 97 % similarity. Thereafter, a representative sequence of each OTU was subjected to taxonomy-based analysis using the Ribosomal Database Project database.

### Gas chromatography–mass spectrometry (GC-MS) analysis of short-chain fatty acids (SCFAs) in plasma

SCFAs in the plasma were quantified by GC-MS (Agilent GC System 7890B, MSD system 5977B) analysis. Briefly, 15 µL of plasma was acidified with 50 µL of 50 % sulfuric acid; 10 µL of 2-ethylbutyric acid as the internal standard and 400 µL of ether were added and the mixture was shaken for 15 min and then centrifuged at 9000 rpm at 4 °C for 10 min. The ether layer was collected and mixed with anhydrous sodium sulfate for dehydration and then detected by GS-MS. One microliter of the sample was injected (split ratio: 5:1) into a straight glass liner and held at 240 °C. Helium (1 mL/min) was used as the carrier gas in a DB-FFAP capillary column (30 m × 0.25 mm × 0.25 μm). The initial oven temperature of 80 °C was maintained for 1 min, increased to 150 °C at a rate of 5 °C/min, and finally raised to 240 °C at a rate of 10 °C/min and held for 12 min. The electron ionization (70 eV and 230 °C) mode was used. All samples, standards, and blanks were randomly analyzed using the GC-MS system.

### Analysis of results

The biochemical parameters and western blotting results were analyzed using one-way ANOVA with Bonferroni post-hoc test. All analyses were performed using Statistical Package for the Social Sciences software. Values are expressed as mean ± standard error of mean and the level of significance was set to *P* < 0.05 for all tests.

## Results

### Weights of maternal and fetal rats, and biochemical variables of maternal rats

The maternal body weight increased in the HFD group, and treatment of the HFD group with metformin (HFMf treatment) significantly reversed this effect. Furthermore, HFMf treatment decreased maternal liver weight gain induced by the HFD. The fetal weight in the HFD group was higher than that in the HFMf treatment and NCD groups. The ALT level in the HFMf treatment group was significantly lower than that in the NCD and HFD groups. The blood sugar level was the highest in HFD maternal rats. There was no significant difference in maternal blood sugar level between the NCD and HFMf groups (Table 1).

**Table 1:** Weights and biochemical variables of the animal subjects in the studied groups

	BW (gm)	Liver (gm)	Liver / BW	Mother net weight (gm)	Fetal weight (gm)	Blood glucose (mg/dL)	AST (U/L)	ALT (U/L)	T-Cholesterol (mg/dL)
**NCD**	**325.6 ± 6.4** ^#^^†^	**9.59 ± 0.4** ^#^	**0.03 ± 0.001** ^†^	**286.2 ± 5.5** ^#^^†^	**2.75 ± 0.26**	**68.83 ± 3.20**	**85.9 ± 9.3**	**34.3 ± 1.1** ^†^	**61.7 ± 5.4**
**HFD**	**395.3 ± 10.9** ^*†^	**12.87 ± 0.5** ^*†^	**0.03 ± 0.001** ^†^	**351.0 ± 11.7** ^*†^	**3.24 ± 0.39**	**77. 88 ± 5.54** ^†^	**98.7 ± 8.2**	**34.7 ± 2.1** ^†^	**60.5 ± 7.1**
**HFMf**	**293.2 ± 9.1** ^*#^	**10.78 ± 0.6** ^#^	**0.04 ± 0.002** ^*#^	**260.1 ± 8.8** ^*#^	**2.56 ± 0.27**	**61.14 ± 3.13** ^#^	**112.3 ± 16.6**	**28.6 ± 1.2** ^*#^	**50.0 ± 6.7**

*NCD* normal-chow diet, *HFD* high-fat diet, *HFMf* high-fat diet co-treated with metformin during pregnancy, *BW* body weight of maternal rats, *Liver* liver weight of the maternal rats, *Mother net weight* calculated by subtracting placental and fetal weights from body weight of maternal rats, *Blood glucose* serum glucose level in the maternal rats, *AST* aspartate transaminase, *ALT* alanine aminotransferase, *T-Cholesterol* serum total cholesterol of maternal rats

^*^p < 0.05 compared with the NCD group

^#^*p* < 0.05 compared with the HFD group

^†^*p* < 0.05 compared with the HFMf group; mean ± standard error

The data were presented as mean ± standard error

### Hematoxylin and eosin staining of maternal liver tissue and intestinal morphology

**Fig. 1:**
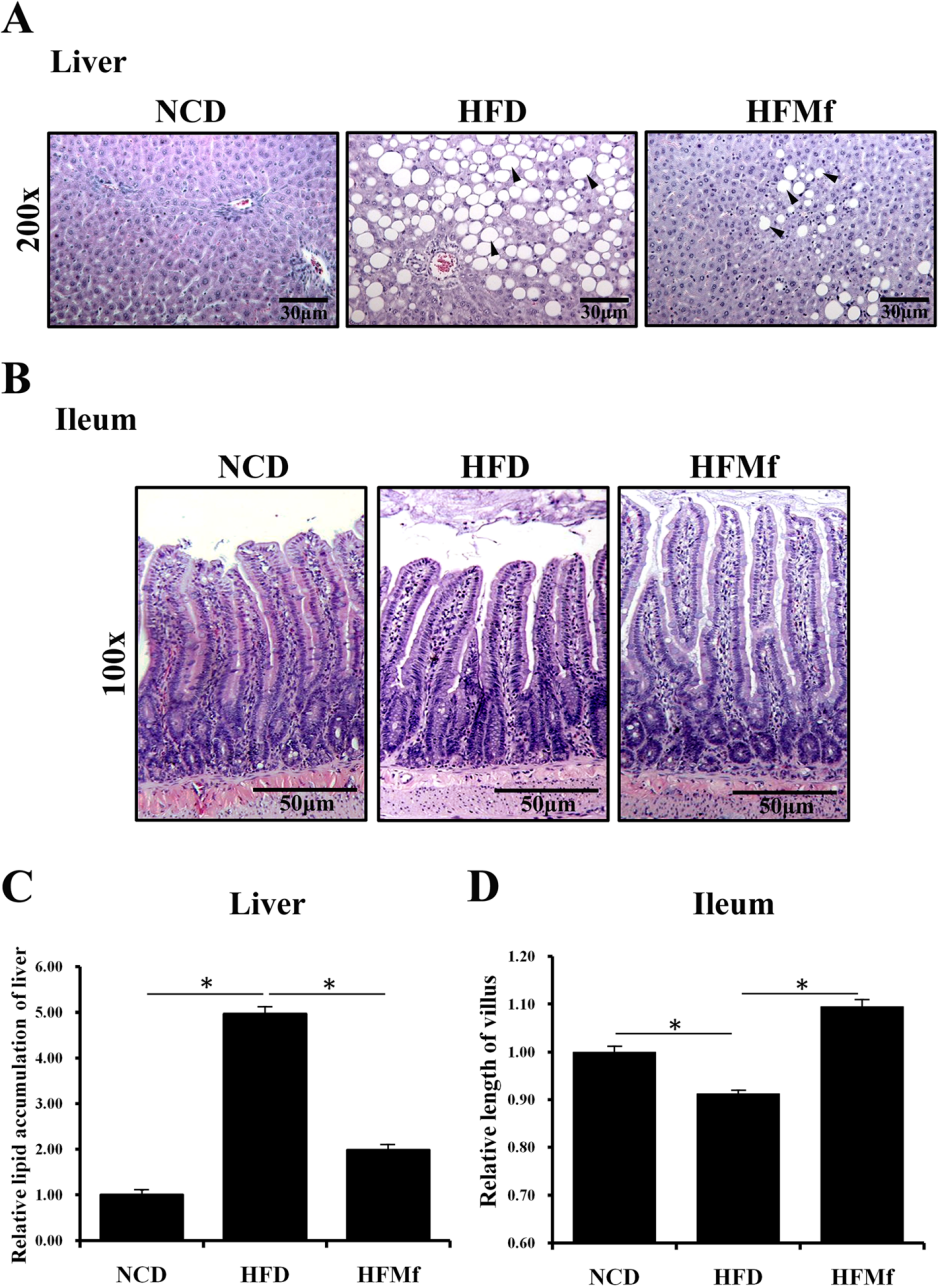
Histological analysis of the maternal liver and ileum. (**a)** H&E staining of the liver and (**b)** ileal tissues. Semi-quantitative analysis of (**c**) hepatic lipid accumulation and (**d**) ileal villous length is shown. NCD: normal-chow diet, HFD: high-fat diet, HFMf: high-fat diet co-treated with metformin during pregnancy (*n* = 6), * *P* < 0.05

Maternal liver steatosis was assessed by H&E staining. The stained liver tissues showed a higher number of lipid droplets in the HFD group than in the NCD group. In addition, compared with the HFD group, the number of lipid droplets in the HFMf group reduced after metformin therapy (Fig. 1a and c). The ileal villous length was significanlty shorter in HFD group than in the NCD and HFMf groups. Prenatal metformin restored the shortening effect of HFD on the ileal villi (Fig. 1b and d).

### Apoptosis analysis in maternal liver and instestinal tissues

**Fig. 2:**
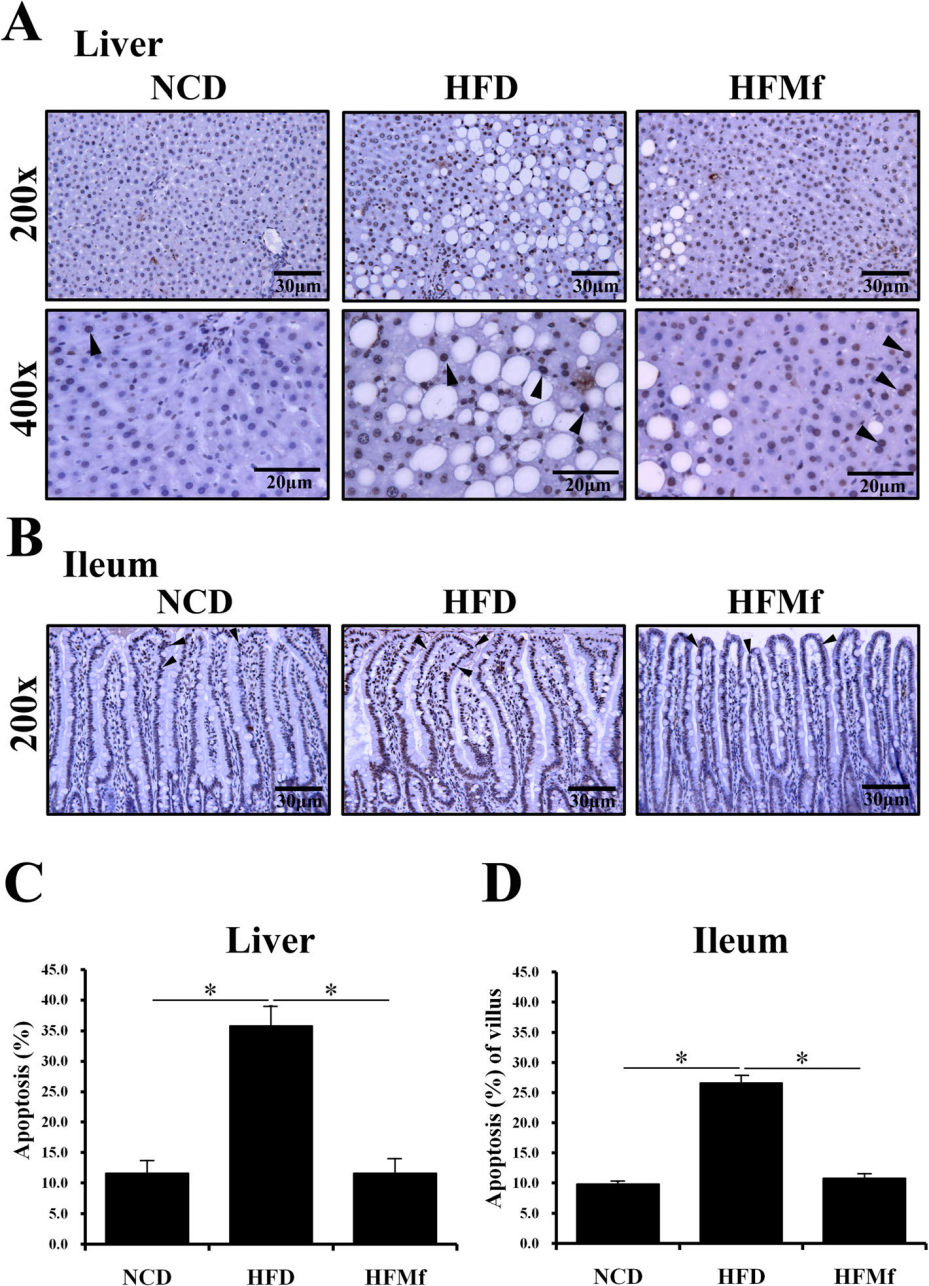
Proportions of apoptotic cells in the maternal liver and ileal villi among different groups. (**a)** TUNEL staining in the liver and (**b)** ileal tissues. TUNEL staining was the strongest in the HFD group. Semi-quantitative analysis of TUNEL-stained cells in (**c**) liver and (**d**) ileal villi is shown. NCD: normal-chow diet, HFD: high-fat diet, HFMf: high-fat diet co-treated with metformin during pregnancy (*n* = 6), * *P* < 0.05

The apoptotic cells were detected by TUNEL staining. Compared with the NCD and HFMf groups, the proportion of apoptotic cells in both maternal liver and ileal tissues was increased in the HFD group (Fig. 2a and b). The results suggested that the HFD increased apoptosis in the maternal liver and intestine, and prenatal metformin administration could sigificantly reduce HFD-induced apoptosis in these tissues (Fig. 2c and d). The rate of cellular apoptosis was over 60 % in the NCD group ileum tissues, which might be due to the effect of anesthesia and cleaning with ice-cold phosphate buffered saline during animal sacrifice.

### Western blotting of ZO-1 and Claudins in the maternal intestine

**Fig. 3:**
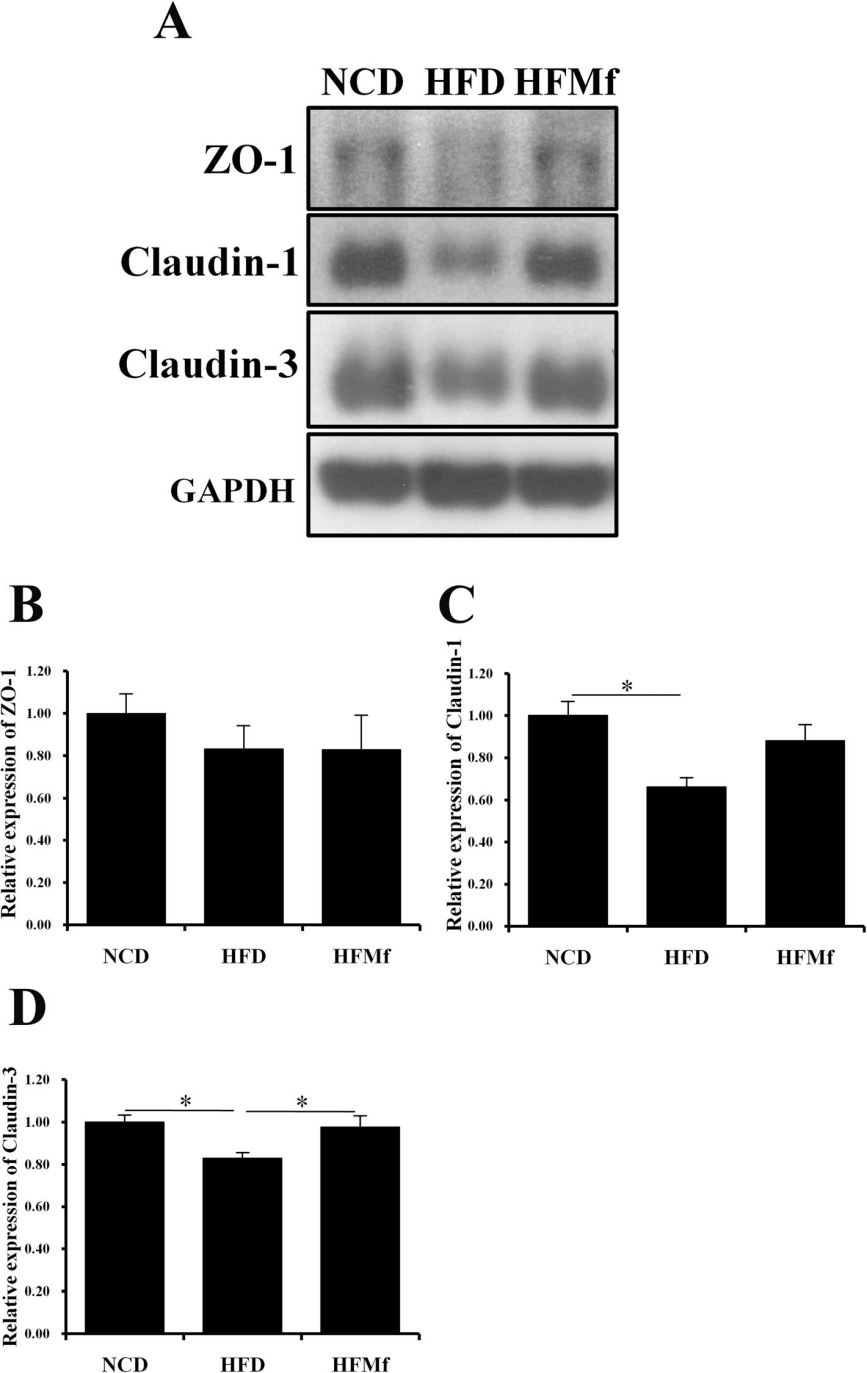
Analysis of tight junction proteins in the intestine of maternal rats. (**a**) Western blotting and semi-quantitative analysis of (**b**) ZO-1, (**c) **claudin-1, and (**d)** Claudin-3. NCD: normal-chow diet, HFD: high-fat diet, HFMf: high-fat diet co-treated with metformin during pregnancy (*n* = 6), * *P* < 0.05

ZO-1, also known as tight junction protein-1, is a 220-kD peripheral membrane protein, which is encoded by the tight junction protein-1 gene in humans. It belongs to the family of zona occludens proteins, which are tight junction-associated proteins, of which, ZO-1 is the first to be cloned [19]. No difference was observed in the expression of ZO-1 after HFD or HFMf treatment (Fig. 3a and b). Several inflammatory disorders are associated with altered expression of tight junction proteins, and especially claudin family members, making them attractive diagnostic and prognostic markers. The Claudin-1 and Claudin-3 levels were reduced in the HFD group compared with that in the NCD and HFMf groups, especially in the Claudin-3 level. Furthermore, the HFMf treatment increased Claudin-3 expression (Fig. 3a, c and d).

### Maternal serum SCFA profile

**Fig. 4:**
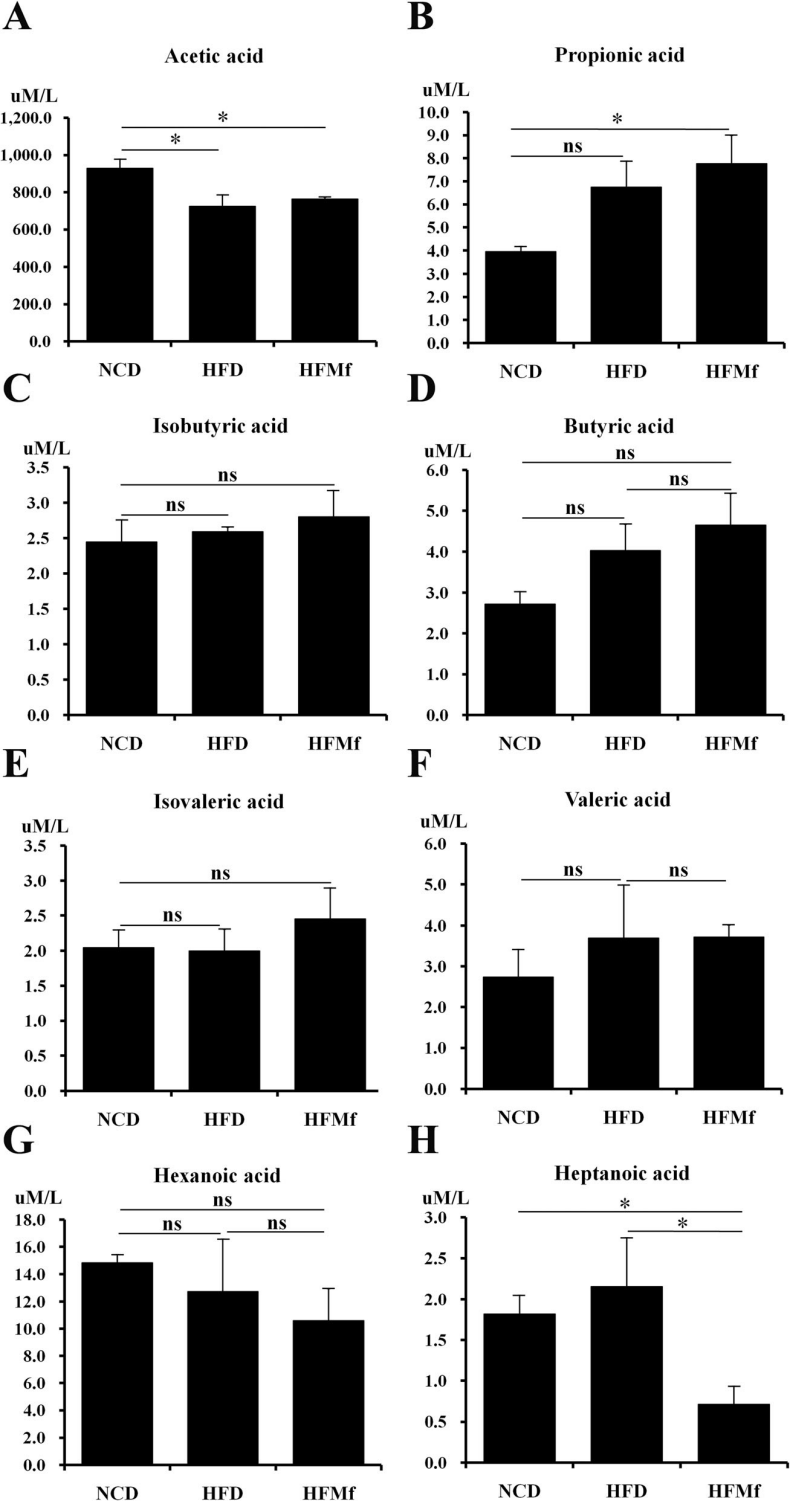
Short-cahin fatty acid profile of maternal rats. Maternal serum levels of (**a**) acetic acid, (**b)** propionic acid, (**c)** isobutyric acid, (**d)** butyric acid,(**e)** isovaleric acid, (**f)** valeric acid, (**g)** hexanoic acid, and (**h)** hepatanoic acid. NCD: normal-chow diet, HFD: high-fat diet, HFMf: high-fat diet co-treated with metformin during pregnancy (*n* = 6), * *P* < 0.05

SCFAs have less than six carbon atoms. Derived from intestinal microbial fermentation of indigestible foods, SCFAs are the main energy source of colonocytes, making them crucial to gastrointestinal health [20, 21]. We analyzed maternal serum acetic acid, propionic acid, isobutyric acid, butyric acid, isovaleric acid, valeric acid, hexanoic acid, and heptanoic acid (Fig. 4). The maternal acetic acid was decreased in the HFD and HFMf groups compared with that in the NCD group (Fig. 4a). The level of maternal propionic acid was higher in the HFMf treatment group than in the HFD and NCD groups (Fig. 4b). There was no significant difference in the isobutyric acid (Fig. 4c), butyric acid (Fig. 4d), isovaleric acid (Fig. 4e), valeric acid (Fig. 4f), and hexanoic acid (Fig. 4g) levels; the level of heptanoic acid in the HFMf treatment group was significantly lower than that in the HFD and NCD groups (Fig. 4h).

### Microbiota of maternal fecal samples

**Fig. 5:**
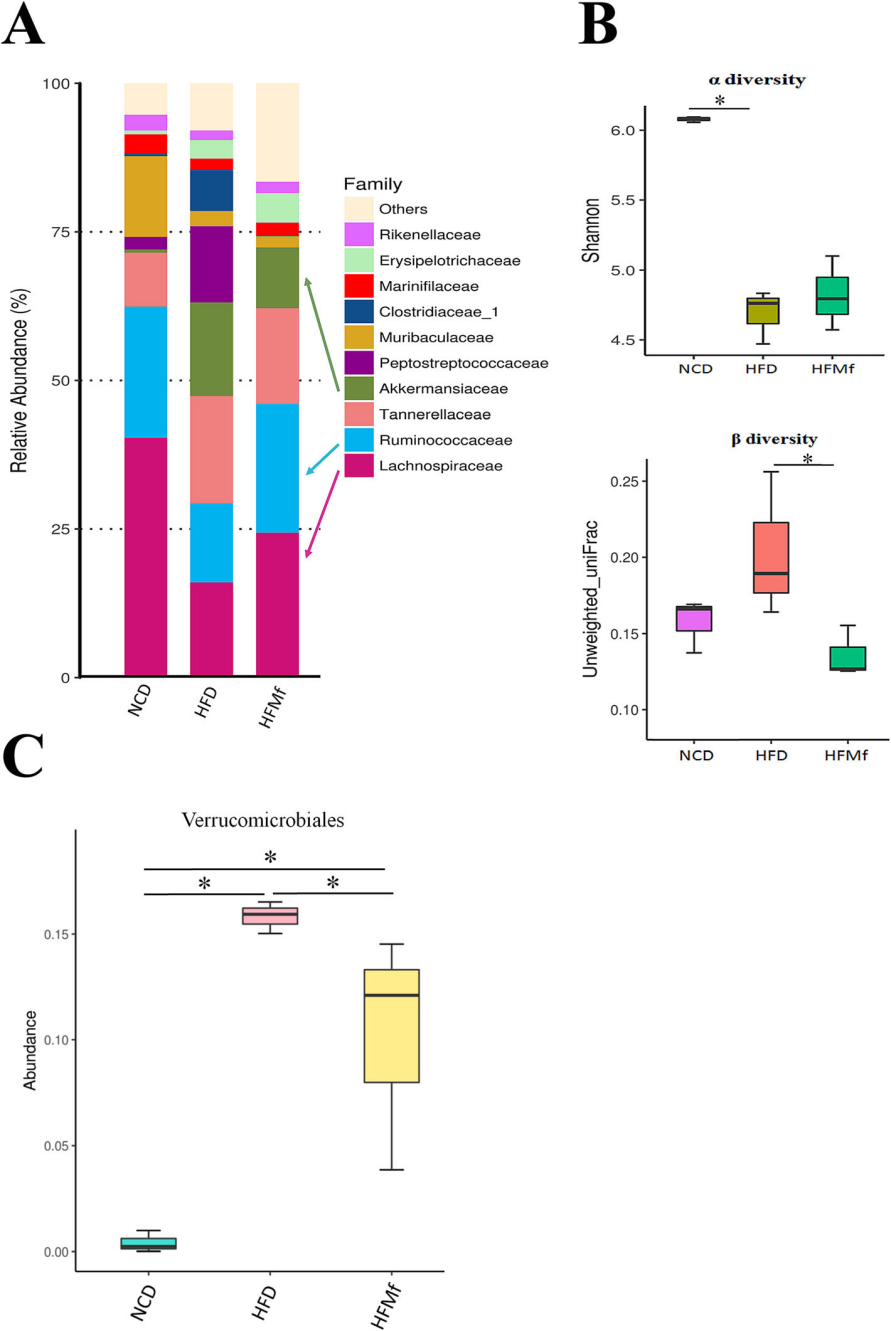
Fecal microbiota of maternal rats (**a**) Composition of the maternal intestinal microbiota at the family level, (**b)** microbial alpha and beta diversities of the maternal intestine, and (**c)** relative abundance of Verrucomicrobia among different groups. NCD: normal-chow diet, HFD: high-fat diet, HFMf: high-fat diet co-treated with metformin during pregnancy (*n* = 6), * *P* < 0.05

The composition of the intestinal microbiota at the family level was different among the three groups (Fig. 5a). HFD significantly reduced the fecal microbiota biodiversity among individual maternal rats, recognized as alpha diversity, in the HFD group compared with that in the NCD group. Prenatal metformin non-significantly increased maternal fecal microbial richness in rats fed HFD. Conversely, the HFD significantly increased the difference in fecal microbiota among individuals, known as beta diverisy, and metformin could ameliorate it (Fig. 5b). The relative abundance of Verrucomicrobia was significanlty higher in the HFD and HFMf groups than in the NCD group. Prenatal metformin obviously reduced the relative abundance of maternal gut Verrucomicrobia increased by HFD (Fig. 5c). Prenatal metformin use could therefore alter the gut microbiota of matneral rats with HFDs.

### Inflammation and apoptosis of fetal tissues: liver and intestine

#### Immunohistochemical (IHC) analysis of Interleukin-6 (IL-6) expression and TUNEL staining of the fetal liver and intestine

**Fig. 6:**
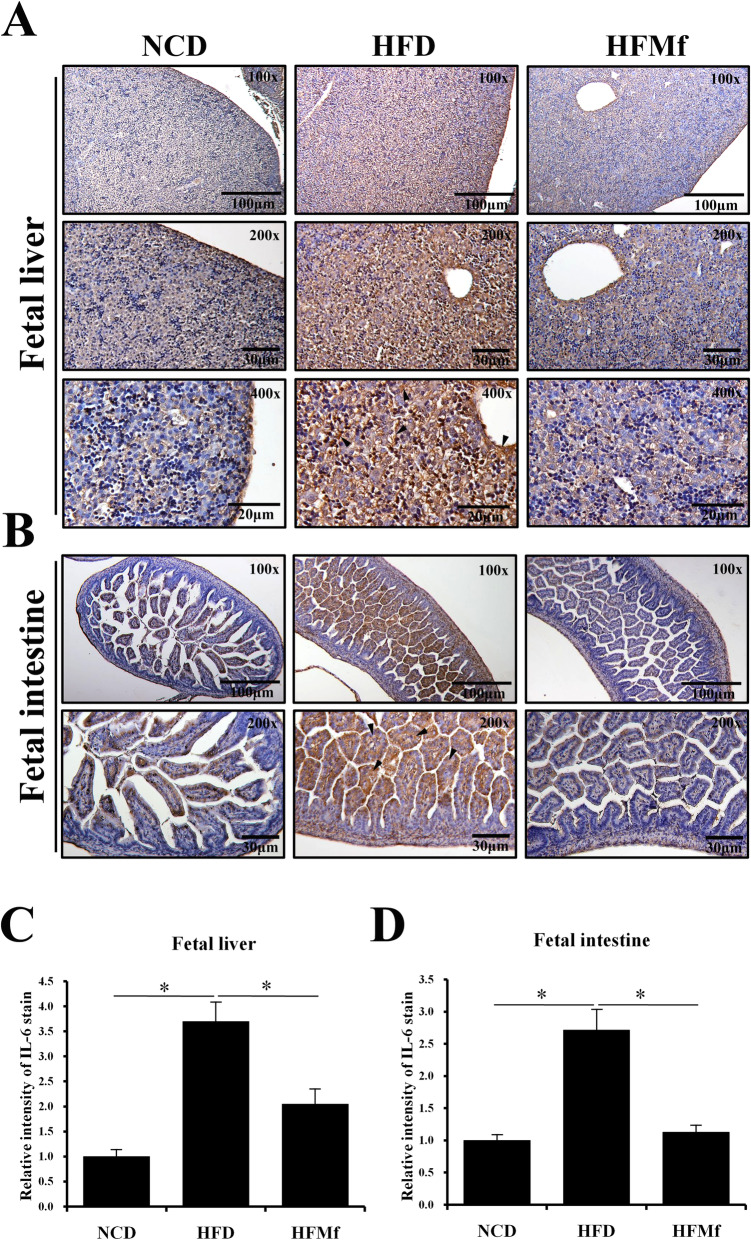
Immunohistochemical analysis of IL-6 expression in the fetal liver and ileum. (**a)** IL-6 staining of the fetal liver and (**b)** fetal ileum. The results of semi-quantitative analysis are shown in (**c**) and (**d**). NCD: normal-chow diet, HFD: high-fat diet, HFMf: high-fat diet co-treated with metformin during pregnancy (*n* = 6), * *P* < 0.05

**Fig. 7:**
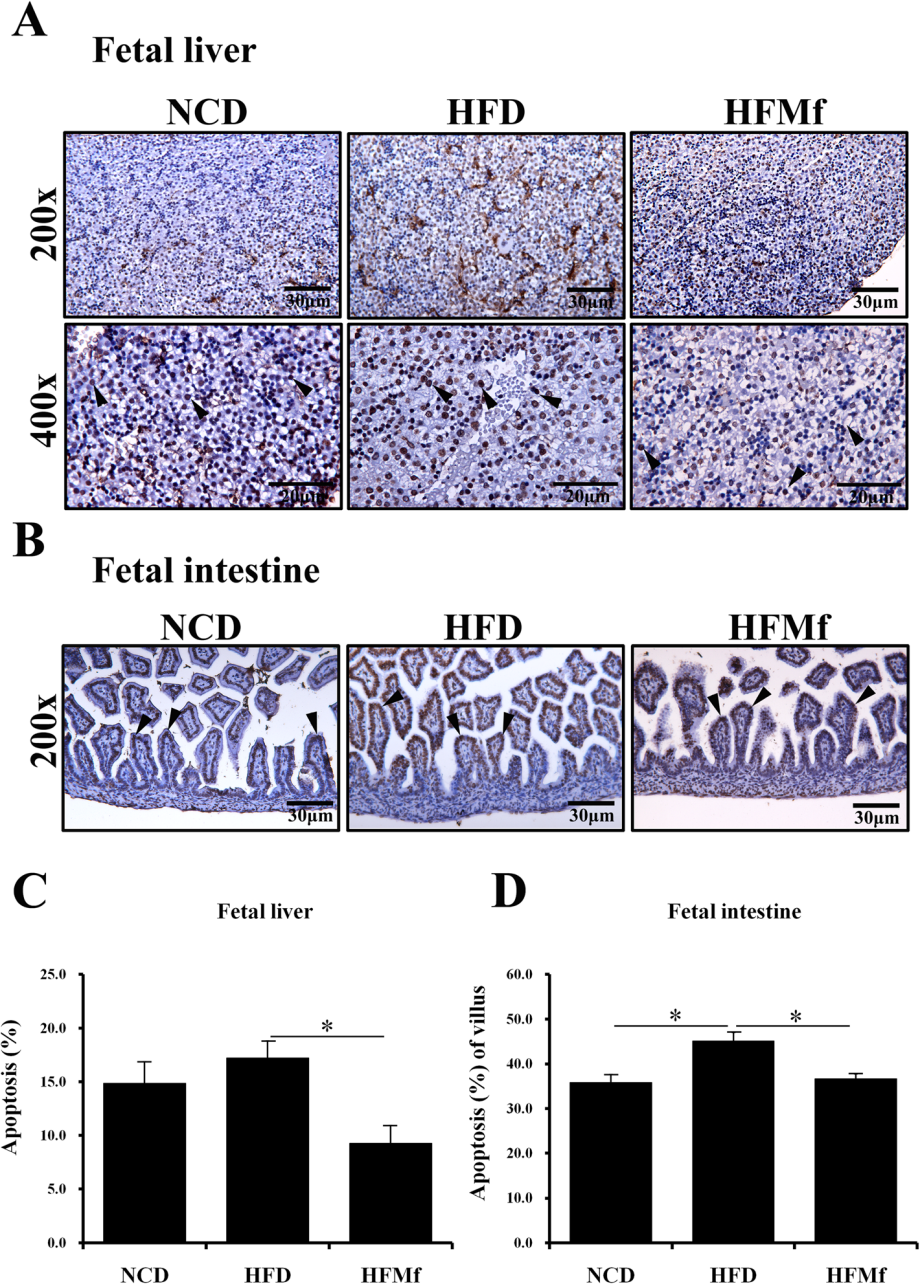
Proportions of apoptotic cells in the fetal liver and ileal villi in among different groups. (**a)** TUNEL staining of the liver and (**b)** ileum. The TUNEL staining was the strongest in the HFD group in both liver and ileum. Semi-quantitative analysis of TUNEL stained cells in the (**c**) liver and (**d**) ileal villi. NCD: normal-chow diet, HFD: high-fat diet, HFMf: high-fat diet co-treated with metformin during pregnancy (*n* = 6), * *P* < 0.05

IL-6 acts as a pro-inflammatory cytokine [22]. IL-6 expression was studied by IHC staining of fetal liver and intestine (Fig. 6a and b). The IL-6 expression in the fetal liver and intestine in the HFD group was significantly higher than that in the NCD and HFMf treatment groups (Fig. 6c and d). The histological analysis revealed that there was no significant shortening of the villous length in the HFD group compared with that in the NCD group. The apoptotic cells were analyzed by TUNEL staining, which was the strongest in the HFD group in both fetal liver and intestine (Fig. 7a and b). The proportion of hepatic apoptotic cells in the HFMf treatment group was significant lower than that in the HFD group (Fig. 7a and c). In the fetal intestine, the proportion of apoptotic cells was significanlty greater in the HFD group than in the NCD and HFMf groups (Fig. 7b and d).

#### Western blotting of inflammation and apoptosis analysis of the fetal liver

**Fig. 8:**
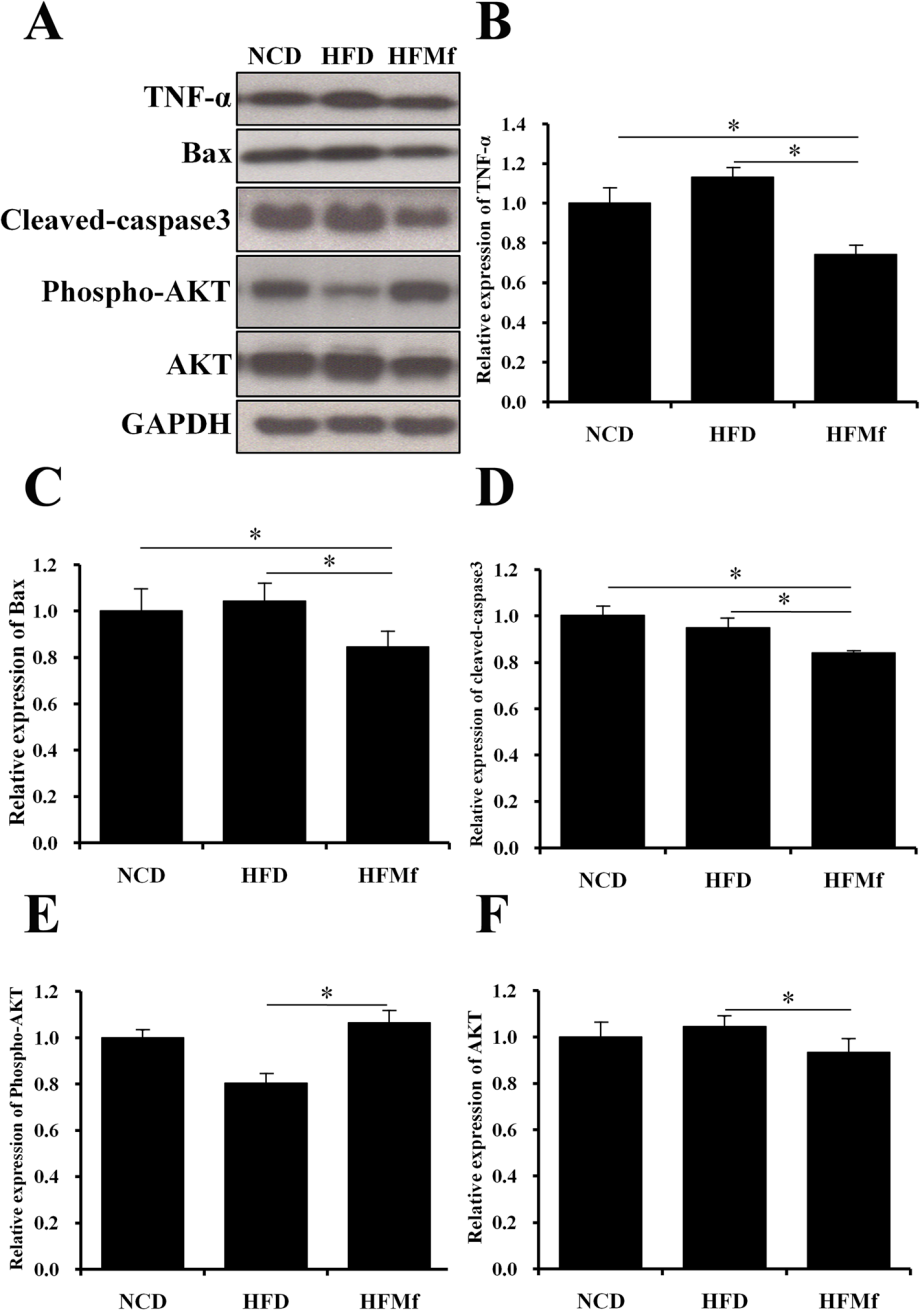
Apoptotic and inflammatory protein expressions in the fetal liver. (**a)** Western blotting analysis showing different fetal liver protein expressions among the three groups. Semi-quantitative analysis of (**b**) TNF-α, (**c)** Bax, (**d)** cleaved caspase-3, (**e)** phospho-AKT, and (**f)** AKT. NCD: normal-chow diet, HFD: high-fat diet, HFMf: high-fat diet co-treated with metformin during pregnancy (*n* = 6), * *P* < 0.05

According to our previous studies, excessive fat accumulation in the liver triggers liver cell inflammation and apoptosis [17]. The expression of the pro-inflammatory cytokine TNF*-*α was the lowest in HFMf treatment group compared with that in the HFD and NCD groups (Fig. 8a and b). AKT, Bax, and caspase-3 have been identified to play roles in liver cell apoptosis [23, 24]. The expression of both Bax and cleaved caspase-3 was significantly decreased in the HFMf treatment group compared with that in the NCD and HFD groups (Fig. 8c and d). The expression of phosphate-AKT in the HFD group was lower than that in the NCD group (though not significant); however, it significantly increased in the HFMf treatment group (Fig. 8a and e). Furthermore, the expression of AKT was higher in the HFD group than in the HFMf treatment group (Fig. 8a and f). There was no significant difference in adenosine monophosphate-activated protein kinase (AMPK) and p-AMPK expressions in the fetus liver among the three groups (data not shown).

## Discussion

The results of this study indicate that prenatal metformin could alleviate HFD-induced obesity and liver steatosis in maternal rats and reverse the adverse effect of maternal HFD on the fetal liver and intestine. It has been reported that metformin could decrease body weight, intrahepatic lipid accumulation, hepatic apoptosis, and serum liver enzyme and serum lipid levels in rodents with liver steatosis [25–28], and these effects were observed in the maternal rats in this study.

The gut microbiota is one of the factors involved in the pathogenesis of NAFLD. Microbiota-derived metabolites such as SCFAs act as molecular signals in the liver. In addition, the increase in intestinal permeability enhances the translocation of microbiota-derived metabolites to the liver via the portal venous system. Dysbiosis and leaky guts induce low-grade inflammation in the liver tissue, leading to liver steatosis [29].

Metformin is considered to change intestinal microbiota and improve many metabolic problems. A study using an HFD mouse model revealed that metformin may reduce bacterial alpha diversity and change the specific microorganisms associated with metabolism [30]. On the contrary, in this study, metformin tended to increase alpha diversity in HFD rats and that metformin could alleviate the differences in the gut microbiota between individual rats fed HFD. The intestinal flora is highly diverse, and is predominantly composed of Firmicutes, Bacteroidetes, and other bacteria including Actinobacteria, Proteobacteria, Verrucomicrobia, and Fusobacteria. Several studies have suggested that the abundance of Verrucomicrobia is associated with obesity and the metabolic syndrome, either inversely or positively [14]. The findings of this study are consistent with those of most studies in mouse models, which showed that high-fat or high-calorie diets increased the abundance of Verrucomicrobia, which is associated with liver steatosis progression [31]. The findings of the present study are also similar to those of a human trial, which showed that the administration of metformin to non-diabetic obese women decreased Verrucomicrobia levels in fecal samples [32].

Previous studies have shown that HFDs reduce small intestinal villous length in a rodent model [33, 34]. In this study, similar results were obtained; furthermore, the decrease in ileal villous length in HFD-maternal rats was reversed by metformin. To the best of our knowledge, this finding has not been reported to date. Xie et al. investigated the effect of HFDs on the intestine of female mice from several aspects and showed that HFDs increased the proportion of apoptotic cells in the colon, but not in the small intestine [34]. In contrast, in the present study, HFDs increased apoptosis in the ileal tissues, and this could be reduced by metformin administration.

Many tight junction proteins, such as the claudin family and ZO-1, maintain the gut barrier and intestinal epithelial homeostasis. Previous studies in mice reported that unlike normal diets, HFDs resulted in the loss of claudin-1, claudin-3, and ZO-1 in the intestinal epithelium [35]. The use of metformin reportedly alleviates the loss of tight junction proteins [36]; this is consistent with the present findings.

Our analysis of the serum SCFA levels in maternal rats revealed that metformin did not increase the level of acetic acid, but increased the level of propionic acid in the serum of HFD-fed maternal rats. These two SCFAs are considered protective factors against NAFLD in a previous study [37]. In summary, the present study findings suggest that metformin affects several aspects of metabolism and reverses the process of liver steatosis.

Furthermore, in this study, the effect of prenatal metformin exposure on the fetal liver and intestines was evaluated. In the fetal intestinal tissues, the expression of IL-6 was significantly higher in the HFD group than in the NCD and HFMf groups. A murine study showed that the intestinal expression of IL-6 is positively correlated with intestinal permeability [38]. The present findings indicate that prenatal metformin probably restores maternal HFD-induced changes in intestinal permeability in the fetus. The present assessment of the proportion of apoptotic cells in the fetal intestine revealed that prenatal metformin significantly alleviated maternal HFD-induced apoptosis in the fetal intestine. This finding has not been reported to date.

The fetal liver tissues were analyzed for IL-6 expression by IHC staining, TUNEL assay, and western blotting to assess the changes in inflammation and apoptosis among different groups. The expression of IL-6 in hepatocytes is positively associated with the degree of inflammation [39]. In this study, prenatal metformin alleviated maternal HFD-induced elevation in IL-6 expression in the fetal liver. A trial on nonhuman primates demonstrated that maternal HFD obviously increases fetal hepatic apoptosis, as determined using TUNEL analysis [40]. Here, the TUNEL analysis of the fetal liver revealed similar results, and clearly suggested that prenatal metformin exerts anti-apoptotic effects on the fetal liver in the present study.

Upregulation of inflammatory mediators, including TNF-α, has been found in NAFLD [41]. In this study, prenatal metformin decreased the TNF-α level in the fetal liver. Analyses of markers related to apoptosis in NAFLD studies have shown an increase in AKT with a decrease in phosphate-AKT, increase in Bax, and increase in cleaved caspase-3 in the hepatic tissue [23, 24, 42]. The present findings showed that the protein expression associated with apoptosis in the HFMf group was significantly lower than that in the HFD and NCD groups, which supported the anti-apoptotic effect of prenatal metformin.

The therapeutic action of metformin against NAFLD is thought to result mainly from the activation of AMPK, and it subsequently inhibits glucogenesis and lipogenesis. Metformin alleviates inflammation and apoptosis in the liver triggered by excess saturated fatty acids by decreasing the deposition of intrahepatic lipids [43]. Furthermore, metformin could change the gut microbiota via the AMPK-independent pathway, thereby ameliorating low-grade inflammation in the intestine [44]. The results of this study are in line with the aforementioned mechanisms and support the effect of prenatal metformin on inflammation and apoptosis in the fetal liver and intestine.

It is well established that maternal obesity and a maternal HFD might result in fetal malprogramming and metabolic morbidity in offspring, such as obesity, metabolic syndromes, and NAFLD. Several animal trials have demonstrated that maternal obesity and HFD contribute to an increase in fetal liver inflammation and fatty liver in offspring [3]. A cohort study in Australian adolescents showed that the diagnosis of female offspring with NAFLD by liver ultrasound at the age of 17 years is independently associated with maternal obesity, whereas this association was not observed in male offspring [45].

In-utero exposure of metformin might influence fetal programming in metabolism via several pathways. One of mechanisms by which metformin reduces maternal hyperglycemia, hyperinsulinemia, and inflammatory cytokines is via the changes in the in-utero environment [46]. In contrast, metformin could freely cross the placenta and have an epigenetic effect on the fetus via AMPK regulations [47], although there was no change in the AMPK level in the fetal stage.

### Comparisons with other studies and what does the current work add to the existing knowledge

Several preclinical studies have suggested prenatal metformin to be an efficient therapy to ameliorate metabolic malprogramming such as hypertension and glucose intolerance in offspring [10, 12, 48]. There were no studies investigated the effect of prenatal metformin in the fetal intestine or anti-apoptotic effect in the fetal liver after maternal HFD. The current study revealed anti-inflammatory and anti-apoptotic effects of prenatal metformin on the fetal intestine and liver. The decrease in ileal villous length in HFD dams was also reversed by metformin.

### Study strengths and limitations

The present animal study demonstrated the beneficial effects of prenatal metformin in reducing inflammation and apoptosis in the fetal liver and intestine, which improves our understanding of the important effects of in-utero metformin exposure on fetal development. However, we did not investigate the effect of prenatal metformin on offspring of obese/HFD mothers and the microbiota in the fetus.

## Conclusions

The present study demonstrated that prenatal metformin could ameliorate the effect of maternal HFD-induced inflammation and apoptosis in the fetal liver and intestine. This beneficial effects of in-utero exposure of metformin on the fetal liver and intestine has not been reported to date. This study supports the use of prenatal metformin in pregnant obese women without diabetes.

## Data Availability

Not applicable.

## Abbreviations

ALT: Alanine transaminase
AST: Aspartate transaminase
AMPK: Adenosine monophosphate-activated protein kinase
DNA: Deoxyribonucleic acid
FFPE: Formalin-fixed, paraffin-embedded
GD: Gestational day
GC-MS: Gas chromatography–mass spectrometry
HFD: High-fat diet
HFMf: Maternal rats under high-fat diet and co-treated with metformin during pregnancy
H&E: Hematoxylin and eosin
IL-6: Interleukin-6
IHC: Immunohistochemical
NAFLD: Non-alcoholic fatty liver disease
NCD: Normal-chow diet
OTU: Operational taxonomic unit
SCFA: Short-chain fatty acid
SD: Sprague-Dawley
TNF-α: Tumor necrosis factor alpha
TUNEL: Terminal deoxynucleotidyl transferase-mediated deoxyuridine triphosphate biotin nick-end labeling
ZO-1: Zonula occludens-1
